# Neoadjuvant chemotherapy does not improve survival for patients with high volume colorectal peritoneal metastases undergoing cytoreductive surgery

**DOI:** 10.1186/s12957-024-03392-8

**Published:** 2024-04-18

**Authors:** Mina Sarofim, Ruwanthi Wijayawardana, Nima Ahmadi, Shoma Barat, Winston Liauw, David L Morris

**Affiliations:** 1 Liver and Peritonectomy Unit, St George Hospital, Sydney, NSW Australia; 2 School of Medicine, University of New South Wales, Sydney, Australia; 3 School of Medicine, University of Sydney, Sydney, Australia; 4 Cancer Care Centre, St George Hospital, Sydney, NSW Australia

**Keywords:** Colorectal cancer, Peritoneal metastases, Cytoreductive surgery, Neoadjuvant chemotherapy, HIPEC

## Abstract

**Background:**

Colorectal peritoneal metastases (CRPM) affects 15% of patients at initial colorectal cancer diagnosis. Neoadjuvant chemotherapy (NAC) prior to cytoreductive surgery (CRS) has been demonstrated to be a safe and feasible option, however there is limited data describing its efficacy in advanced peritoneal disease. This study evaluated the effect of NAC on survival in patients with high volume CRPM undergoing CRS with or without HIPEC.

**Methods:**

A retrospective review of all patients who underwent CRS with or without HIPEC for CRPM from 2004 to 2019 at our institution was performed. The cohort was divided based on peritoneal carcinomatosis index (PCI) at surgery: Low Volume (PCI ≤ 16) and High Volume (PCI > 16).

**Results:**

A total of 326 patients underwent CRS with HIPEC for CRPM. There were 39 patients (12%) with High Volume disease, and 15 of these (38%) received NAC. Patients with High Volume disease had significantly longer operating time, lower likelihood of complete macroscopic cytoreduction (CC-0 score), longer intensive care unit length of stay and longer hospital stay compared to Low Volume disease. In High Volume disease, the NAC group had a significantly shorter median survival of 14.4 months compared to 23.8 months in the non-NAC group (*p* = 0.046).

**Conclusion:**

Patients with High Volume CRPM achieved good median survival following CRS with HIPEC, which challenges the current PCI threshold for offering CRS. The use of NAC in this cohort did not increase perioperative morbidity but was associated with significantly shorter median survival compared to upfront surgery.

## Introduction

Colorectal cancer (CRC) is the third most common cancer globally, with metastatic disease representing the principal cause of mortality [1, 2]. Transcoelomic spread from the bowel to involve the peritoneal epithelium is termed peritoneal metastasis, which represents advanced localised spread rather than true distant metastases. Colorectal peritoneal metastases (CRPM) is present in up to 15% of patients at initial diagnosis, and up to 20% in those who develop recurrence [3, 4]. CRPM dramatically reduces overall survival by 30–40% [5]. 

Systemic chemotherapy in the absence of surgery was the traditional treatment option for CRPM but offered a median survival of 3–7 months [6–8]. Over the last three decades, the paradigm of surgical management has shifted from palliative measures for symptom relief to aggressive, potentially curative treatment [9, 10]. The optimal operative approach is cytoreductive surgery (CRS) with or without hyperthermic intraperitoneal chemotherapy (HIPEC). Combined with systemic chemotherapy, patients now have a 3-year survival of over 50% and median survival of 41 months [7, 10–15]. Peritoneal carcinomatosis index (PCI) is a score out of 39 which reflects the volume of CRPM and is the major prognostic factor for long-term survival. Various centres use strict PCI thresholds – ranging from 10 to 20 – above which CRS is contra-indicated due to increased morbidity and lack of survival benefit [15–17]. Ability to achieve a complete macroscopic cytoreduction (CC-0 score) is also significantly linked to improved prognosis [10, 18]. 

The rapidly evolving use of neoadjuvant chemotherapy, that is prior to surgery, has shown promising results in improving survival or down-staging locally advanced tumours of the rectum, pancreas, urothelium and breast [19–22]. Neoadjuvant chemotherapy for CRPM has been demonstrated to be a safe and feasible option, however there is conflicting evidence to support its efficacy. Confounding factors of heterogenous patient selection, small cohort studies, diversity in chemotherapy regimens, and most significantly the focus on low volume CRPM complicate the establishment of high-quality recommendations or their generalisability to patients with relatively advanced peritoneal disease [13, 23–25]. The primary aim of this study was to evaluate whether neoadjuvant chemotherapy improves survival in patients with high volume CRPM undergoing CRS. The secondary aim was to re-evaluate the existing PCI threshold used to determine suitability for CRS with or without HIPEC.

## Methods

### Study design

A retrospective cohort study was performed in a tertiary, high-volume peritoneal malignancy unit in Sydney Australia from February 2004 to February 2019. This study was designed to align with the Strengthening the Reporting of Observational Studies in Epidemiology (STROBE) guidelines [26]. 

### Participants

Records of all patients who underwent CRS with HIPEC for CRPM at our institution were retrospectively reviewed from a prospectively maintained database. To compare the impact of neoadjuvant chemotherapy, the cohort was divided into two groups based on volume of disease at surgery: PCI ≤ 16 (Low volume) and PCI > 16 (High volume). This cut-off was selected as it represents a significant burden of disease for which some centres may not offer CRS [27]. 

The research protocol obtained ethics approval from the local health district as low/negligible risk.

### Variables

Collected data included patient demographics, functional status, CRC location and original diagnosis date, chemotherapy regimen, length of stay, peritoneal carcinomatosis index (PCI), completeness of cytoreduction (CC) score, perioperative morbidity defined by Clavien-Dindo Classification [28] Grade III (complication requiring surgical, endoscopic or radiological intervention) or Grade IV (complication requiring intensive care or organ support), and follow up data.

Patients who were allocated to receive neoadjuvant chemotherapy prior to CRS, the type of regimen (typically FOLFOX, FOLFIRI and/or Bevacizumab), and subsequent adjuvant treatment was either decided by our multi-disciplinary surgical oncology meeting based on an individualised case-by-case review of the clinical, histopathological and radiological presentation, or some patients were referred to our tertiary unit having already commenced systemic treatment based on local surgical oncology consensus. Patients who progressed on neoadjuvant treatment did not proceed to CRS.

### Outcomes

Survival was defined as time from date of CRS with or without HIPEC until death. Persons not marked as dead during follow up were allocated a status of alive and included in survival calculations. Patients marked as ‘lost to follow up’ were censored from the overall population group at that time point.

### Statistical methods

Statistical analysis was done with SPSS version 24 (IBM®, USA). Mean with corresponding standard deviation [SD] for normal distribution data, or median with corresponding range for non-normal distribution data were determined as appropriate. Univariate and multivariate analysis was carried out for continuous and categorical variables. The significance value was *p* < 0.05. Cox regression method for proportional hazard ratio was used to measure survival probability at given time calculated as part of the hazard function at time. Kaplan-Meier technique was then utilised to plot the survival curve and compare outcome between groups.

## Results

### Participants

A total of 326 patients underwent CRS with HIPEC for CRPM. There were 111 patients who received neoadjuvant chemotherapy (NAC), and 215 patients proceeded straight to surgery (non-NAC). There were 39 patients (12%) with High Volume disease (PCI > 16) and 15 of these (38%) received NAC. All were metachronous cases.

### Descriptive data

Mean age of the entire cohort was 56 years old, and male patients made up 40.8%. Overall perioperative mortality rate was 0.92%. Patient characteristics are summarised in Table 1. Between NAC and non-NAC groups, there was no significant difference in age, gender or functional status based on American Society of Anaesthesiologist (ASA) classification or Eastern Cooperative Oncology Group (ECOG) status. Location of the primary CRC tumour was right sided (ascending/transverse colon), left sided (descending/sigmoid colon) or rectal in a similar proportion between these two groups (*p* = 0.29). Pre-operative tumour marker values were not significantly different. Median surgical PCI was also not significantly different (*p* = 0.15), and optimum CC-0 score was similarly achieved in 93.7% and 95.8% respectively (*p* = 0.14). HIPEC (either Mitomycin C or Oxaliplatin) was used in 88.3% and 91.6% in the NAC and non-NAC groups respectively (*p* = 0.38).

**Table 1:** Cohort characteristics between neoadjuvant chemotherapy (NAC) and non-neoadjuvant chemotherapy (non-NAC) groups

	NAC	Non-NAC	p-value
**Number [%]**	111 [34]	215 [66]	
Age (mean years [SD])	54.5 [13.9]	56.7 [14.2]	0.23
Male (%)	42	40	0.49
ASA (median [range])	3 [0–4]	3 [0–4]	0.10
ECOG (median [range])	1 [0–2]	1 [0–3]	0.06
**Primary tumour location**			0.29
Right colon (n [%])	48 [46.2]	91 [48.7]	
Left colon (n [%])	41 [39.4]	62 [33.2]	
Rectum (n [%])	15 [14.4]	34 [18.2]	
**Tumour markers**			
CEA (mean [SD])	45 [248]	29 [167]	0.10
CA 19.9 (mean [SD])	45 [91]	60 [272]	0.69
CA 125 (mean [SD])	22 [39]	38 [108]	0.16
**Operative outcome**			
PCI (median [range])	9 [1–39]	8 [1–35]	0.15
CC-0 score (n [%])	104 [93.7]	206 [95.8]	0.14
HIPEC (n [%])	98 [88.3]	197 [91.6]	0.38
Morbidity~ (n[%])	33 [29.7]	68 [31.6]	0.82
**Perioperative mortality (n [%])**	1 [0.9]	2 [0.93]	0.97

ASA = American Society of Anaesthesiologist classification; ECOG = Eastern Cooperative Oncology Group status; PCI = Peritoneal Carcinomatosis Index; CC = completeness of cytoreduction; HIPEC = hyperthermic intraperitoneal chemotherapy

$\sim$Based on Clavien-Dindo Grade III or IV

Comparison between patients in Low and High Volume groups showed no significant difference in baseline characteristics (Table 2). The pre-operative tumour markers CA19.9 and CA125 were significantly higher in the High Volume group. CC-0 score was achieved significantly more often in the Low Volume group, 95.5% versus 71.8% (*p* < 0.01). Significantly longer operating time (*p* < 0.01), longer Intensive Care Unit length of stay (*p* < 0.01) and longer total hospital stay (*p* = 0.02) occurred in the High Volume group. Perioperative morbidity also occurred significantly more frequently in this group (*p* < 0.01).

Among patients with High Volume disease, comparison between NAC and non-NAC groups revealed no difference in age, gender or functional status. Preoperative tumour markers were not significantly different, nor was achievement of CC-0 score, operating time, hospital stay or perioperative morbidity (Table 3).

**Table 2:** Patient characteristics between Low Volume and High Volume groups

	Low Volume	High Volume	p-value
**Number [%]**	287 [88]	39 [12]	
Age (mean years [SD])	56.4 [13.2]	51.4 [16.7]	0.65
Male (%)	39	57	0.39
ASA (median [range])	3 [0–4]	3 [0–4]	0.38
ECOG (median [range])	1 [0–3]	1 [0–2]	0.63
**Tumour markers**			
CEA (mean [SD])	31 [93]	79 [131]	0.22
CA19.9 (mean [SD])	28 [159]	35 [51]	< 0.01
CA125 (mean [SD])	42 [109]	177 [637]	0.01
**Operative outcome**			
PCI (median [range])	8 [1–16]	20 [17–39]	< 0.01
CC-0 score (%)	95.5	71.8	< 0.01
HIPEC (%)	87.5	89.7	0.11
Operating time (mean hours [SD])	6.6 [2.5]	8.7 [3.5]	< 0.01
ICU LOS (mean days [SD])	2.3 [1.9]	8.4 [18.6]	< 0.01
Hospital LOS (mean days [SD])	21.3 [26.4]	31.5 [31.6]	0.02
Morbidity~ (%)	28.6	35.9	< 0.01
**Perioperative mortality (n[%])**	2 [0.7]	1 [2.5]	0.96

ASA = American Society of Anaesthesiologist classification; ECOG = Eastern Cooperative Oncology Group status; PCI = Peritoneal Carcinomatosis Index; CC = completeness of cytoreduction; HIPEC = hyperthermic intraperitoneal chemotherapy; ICU = intensive care unit; LOS = length of stay

$\sim$Based on Clavien-Dindo Grade III or IV

**Table 3:** Description and outcome of patients with High Volume disease based on neoadjuvant chemotherapy (NAC) status

	NAC	Non-NAC	p-value
**Number [%]**	15 [38]	24 [62]	
**Tumour markers**			
CEA (mean [SD])	50 [57]	34 [50]	0.55
CA19.9 (mean [SD])	74 [113]	285 [835]	0.98
CA125 (mean [SD])	77 [95]	123 [160]	0.95
**Operative outcome**			
PCI (median [range])	20 [17–29]	20 [17–35]	0.31
CC-0 score (%)	69.7	79.1	0.44
HIPEC (%)	80.0	79.1	0.21
Operating time (mean hours [SD])	9.9 [3.7]	8.0 [3.3]	0.07
ICU LOS (mean days [SD])	12.3 [27.6]	6.2 [10.5]	0.22
Hospital LOS (mean days [SD])	37.0 [41.8]	28.3 [24.3]	0.24
Morbidity~ (%)	33.3	37.5	0.79
**Perioperative mortality (n[%])**	0 (0)	1 (4.2)	0.89

PCI = Peritoneal Carcinomatosis Index; CC = completeness of cytoreduction; HIPEC = hyperthermic intraperitoneal chemotherapy; ICU = intensive care unit; LOS = length of stay

$\sim$Based on Clavien-Dindo Grade III or IV

### Survival data

Patients with High Volume disease who received NAC had a significantly shorter median survival, 14.4 months versus 23.8 months (*p* = 0.046) compared to non-NAC. In patients with Low Volume disease, those who received NAC had shorter median survival but this was not significantly different, 36.5 months versus 46.4 months (*p* = 0.17).

**Fig. 1:**
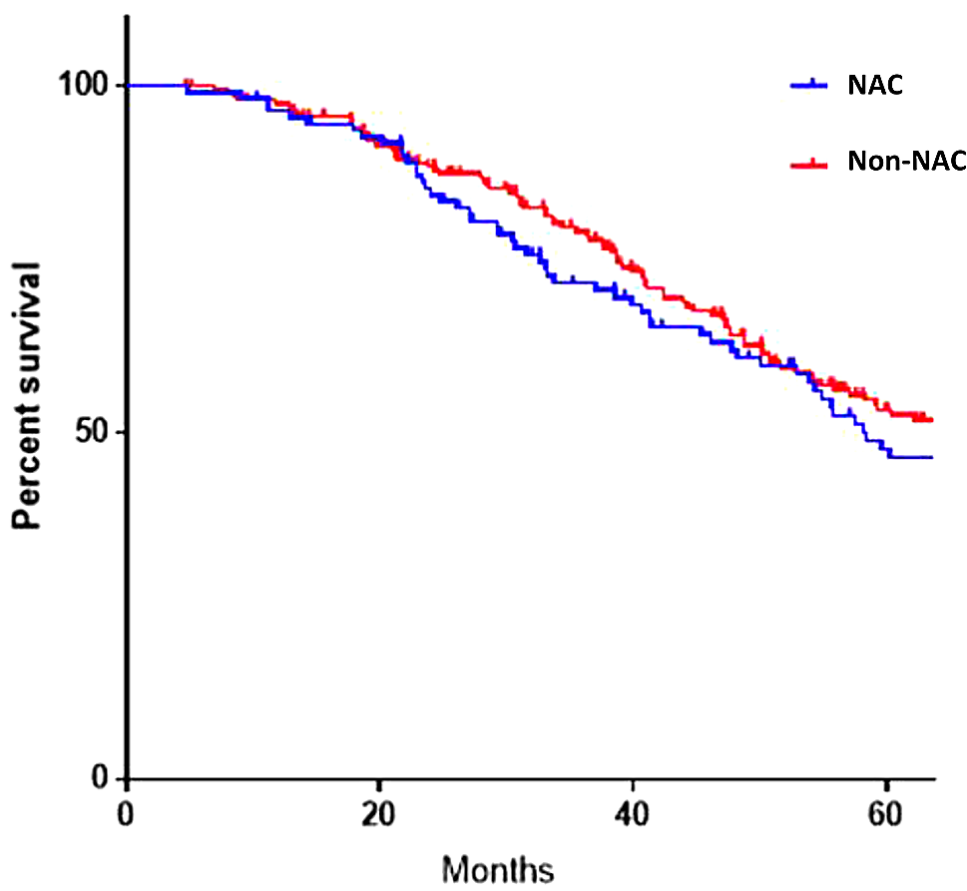
Kaplan Meier survival curve of the total cohort between NAC (blue) and non-NAC (red) patients

**Fig. 2:**
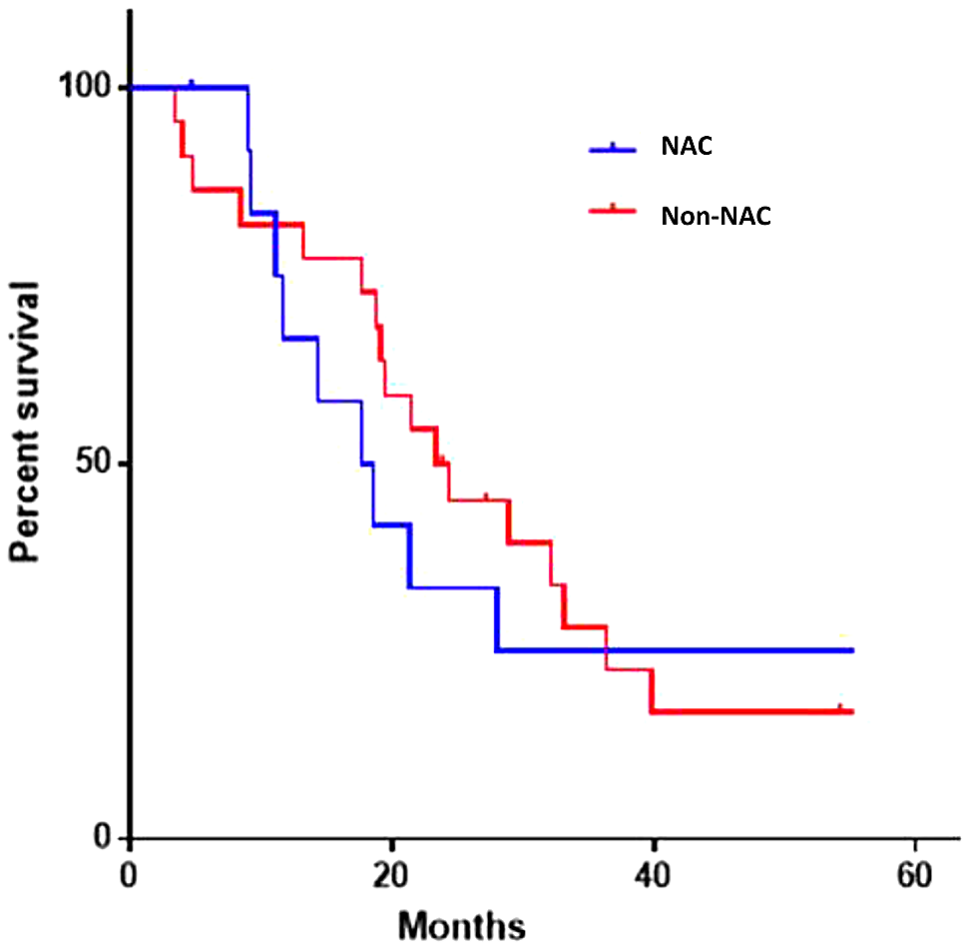
Kaplan Meier survival curve of high volume group between NAC (blue) and non-NAC (red) patients

The survival probability was analysed with Kaplan Meir curves between NAC and non-NAC patients in the total cohort (Fig. 1), and within the High Volume cohort (Fig. 2). The estimated 2-year survival in patients with High Volume disease given NAC was 38.4% compared to 59% in the non-NAC group.

### Other analyses

Primary tumour location was not significantly correlated to survival in either Low or High Volume disease. There was also no significant survival difference if Mitomycin C or Oxaliplatin was used for HIPEC in either group.

## Discussion

This study contributes seminal evidence evaluating the role of neoadjuvant chemotherapy in CRS for CRPM, which is currently lacking for patients with high PCI. Importantly, patients with High Volume disease achieved meaningful median survival following CRS and HIPEC which challenges the current status quo. Unsurprisingly however, it was associated with increased operating time, lower likelihood of complete macroscopic cytoreduction, and increased perioperative complications compared to Low Volume disease. Neoadjuvant chemotherapy in High Volume patients was associated with a reduction in long-term survival, compared to those who proceeded straight to CRS with HIPEC. Across our entire cohort, neoadjuvant chemotherapy did not increase perioperative morbidity or mortality.

Current international consensus recommends the use of CRS and HIPEC in experienced units for fit patients with PCI less than 16 if complete cytoreduction is achievable. Some institutions may consider PCI thresholds up to 20, as beyond this 5-year survival is reported 0–12% [16, 17, 27]. The fundamental rationale for CRS and HIPEC is that peritoneal metastases represent locoregional spread rather than systemic disease. This is nicely demonstrated in a study of 15 patients with High Volume disease – which they defined as PCI ≥ 16 – where CRS and HIPEC successfully achieved 2-year peritoneal disease-free survival comparable to Low Volume patients. They also report a median survival of 14 months in their High Volume group [29]. More recently, a study evaluating CRS and HIPEC in 43 patients with extremely High Volume (PCI ≥ 20) concluded that it was to achieve similar survival to their Low Volume counterparts [30]. When combined with the survival data of our High Volume non-NAC group, we propose that CRS with HIPEC should be offered to well selected patients more liberally rather than exclusion based on strict adherence to a PCI cut-off. The acceptable perioperative morbidity and mortality rates demonstrated are well within the values published in the literature [31]. As peritoneal malignancy surgery continues to evolve, PCI thresholds will continually be tested, particularly for patients facing limited non-curative systemic options [32]. 

A recent review by Flood et al. [24] proposes a mechanism by which neoadjuvant chemotherapy was able to produce better 5-year survival in CRS for CRPM compared to upfront surgery in their meta-analysis. Tumour downstaging or elimination of micrometastatic disease are very plausible and have been seen in various other tumour entities [19–22]. However the median PCI range in their 12 included studies (predominantly retrospective, low-quality) was 5–14, which would be considered Low Volume disease based on our grouping. As is much of the existing literature, their results are not generalisable to a cohort with a disease burden above the upper limit of international consensus. Therefore it may be deduced that unlike patients with low PCI, neoadjuvant chemotherapy in patients with high PCI does not improve long-term survival but rather postpones the beneficial effect of CRS with or without HIPEC. Furthermore, favourable results of upfront surgery have previously challenged the need for neoadjuvant chemotherapy altogether [33]. The only existing phase III randomised clinical trial is CAIRO6 which has so far demonstrated comparability to upfront CRS with HIPEC in phase II data [34]. 

Exactly why neoadjuvant treatment is associated with poorer survival is difficult to authoritatively answer, but there may be several contributing factors. Firstly, preoperative chemotherapy by its very nature will delay definitive CRS and HIPEC which is the cornerstone of management. Secondly, deconditioning associated with cytotoxic medications impairs physical fitness and nutrition prior to radical abdominal surgery [35]. Thirdly, the anaesthetic stress response in the perioperative period has long been hypothesised to increase the likelihood of cancer dissemination and metastasis which may be compounded in a systemically pre-treated patient [36]. Fourthly, intra-tumoural heterogeneity and clonal expansion after neoadjuvant treatment may result in resistance to further systemic treatment due to DNA repair mechanisms in the prevalent cell lines which reverse intended drug-induced damage. This concept was also suggested to account for the absence of survival benefit of HIPEC in the phase III trial (PRODIGE 7) which randomised neoadjuvant treated patients to receive CRS with HIPEC versus CRS alone [37]. From a pragmatic viewpoint on the other hand, neoadjuvant chemotherapy challenges tumour biology, which may not be fully reflected in existing synaptic reporting. Response to systemic treatment is used as a surrogate prognostic marker of favourable disease phenotype which should proceed to CRS, although this is not strictly evidence-based [38]. Disease progression in this setting, particularly in extra-abdominal locations, provides valuable information that CRS would be a futile treatment.

Oncological attempts to improve survival have also occurred alongside surgical advances of CRS, as evidenced by the investigation of neoadjuvant FOLFOX in locally advanced CRC [39]. The use of adjuvant chemotherapy was not the focus of this study, but continues to be commonly given, regardless of neoadjuvant chemotherapy status. Bevacizumab, an antivascular endothelial growth factor antibody, is used in over 40% of patients receiving neoadjuvant chemotherapy. Initial fears of increased wound infection or anastomotic leak have been overcome [24]. The identification that some peritoneal tumours contain a mucinous component helps explain their poor drug penetrance and increased resistance to systemic treatment. In vitro studies of mucolytic therapy (bromelain and acetylcysteine) have demonstrated cytotoxicity against colorectal carcinoma cell lines, as well as producing a synergistic potentiation of other cytotoxic agents – a promising avenue for future systemic or intraperitoneal use [40]. Additionally, patient-derived tumour organoids are an emerging tool in precision oncology. They can be used as ex vivo study models that preserve the original tumour microenvironment, act as biomarkers, and generate drug efficacy data to predict response to cytotoxic therapy [41]. 

Although this study was performed in a specialist peritoneal malignancy unit, we acknowledge several limitations. Firstly the retrospective design and secondly the small High Volume sample size (total 39) mandate cautious interpretation. The relative rarity of this surgical condition, combined with the current level of equipoise regarding both neoadjuvant chemotherapy and surgical management in High Volume disease, have resulted in a lack of high-quality randomised or prospective studies to compare results [34, 42]. A future multi-institutional study with a larger cohort of patients and homogenous NAC regime will be helpful to refine the exact role of NAC. A third limitation is the inherent selection bias of the non-randomised allocation to NAC or non-NAC groups which limits generalisability to healthcare settings with different treatment protocols. Fourthly, survival time (from surgery) in the NAC group does not include the chemotherapy duration; while the inclusion of these extra pre-operative months of treatment may have narrowed the gap in median survival from time of diagnosis, it is unlikely that it would have accounted for the entire median 10 month difference. As such, it is unlikely neoadjuvant treatment had any positive benefit in our cohort of High Volume patients. This study would be strengthened by availability of genetic analysis of tumours (such as microsatellite instability, KRAS and BRAF mutations), or specific details regarding adjuvant chemotherapy use, side-effect profile and completion which may be potential confounders. The addition of tumour regression grading would allow us to determine histopathological differences between those who did and did not respond to neoadjuvant treatment.

## Conclusion

Neoadjuvant chemotherapy had no associated increase in perioperative morbidity. Those with High Volume CRPM experience increased operating time, lower likelihood of CC-0 score, and longer length of hospital stay. Patients with High Volume CRPM who receive neoadjuvant chemotherapy had significantly shorter median survival post CRS and HIPEC, but those who proceed straight to surgery achieved respectable median survival. This challenges the existing PCI thresholds used to determine suitability for CRS and that neoadjuvant treatment should be used cautiously in High Volume CRPM.

## Data Availability

All data analysed during this study are available upon reasonable written request to the corresponding author.
